# Optimal metal domain size for photocatalysis with hybrid semiconductor-metal nanorods

**DOI:** 10.1038/ncomms10413

**Published:** 2016-01-19

**Authors:** Yuval Ben-Shahar, Francesco Scotognella, Ilka Kriegel, Luca Moretti, Giulio Cerullo, Eran Rabani, Uri Banin

**Affiliations:** 1 The Institute of Chemistry and Center for Nanoscience and Nanotechnology, The Hebrew University of Jerusalem, Edmond Safra Campus Givat-Ram, Jerusalem 91904, Israel; 2 Dipartimento di Fisica, IFN-CNR, Politecnico di Milano, 20133 Milan, Italy; 3 Department of Chemistry, University of California and Lawrence Berkeley National Laboratory, Berkeley, California 94720-1460, USA; 4 The Sackler Institute for Computational Molecular and Materials Science, Tel Aviv University, Tel Aviv 69978, Israel

## Abstract

Semiconductor-metal hybrid nanostructures offer a highly controllable platform for light-induced charge separation, with direct relevance for their implementation in photocatalysis. Advances in the synthesis allow for control over the size, shape and morphology, providing tunability of the optical and electronic properties. A critical determining factor of the photocatalytic cycle is the metal domain characteristics and in particular its size, a subject that lacks deep understanding. Here, using a well-defined model system of cadmium sulfide-gold nanorods, we address the effect of the gold tip size on the photocatalytic function, including the charge transfer dynamics and hydrogen production efficiency. A combination of transient absorption, hydrogen evolution kinetics and theoretical modelling reveal a non-monotonic behaviour with size of the gold tip, leading to an optimal metal domain size for the most efficient photocatalysis. We show that this results from the size-dependent interplay of the metal domain charging, the relative band-alignments, and the resulting kinetics.

## Abstract (web summary)

The efficiency of photocatalysis on semiconductor-metal hybrid nanostructures can be influenced by myriad factors. Here, through both experimental and theoretical efforts, the authors elucidate the influence of metal domain size upon performance of such structures, allowing future rational design.

## Graphical abstract

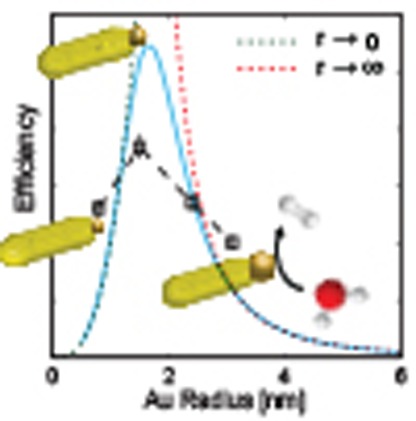

The synergistic optical and chemical properties of semiconductor-metal hybrid nanoparticles lead to light-induced charge separation^1^,^2^, opening the path for their function as photocatalysts in redox reactions, including hydrogen generation by water splitting^3^,^4^,^5^,^6^,^7^, generation of radicals and photodegradation of organic contaminants^8^. The complexity of the photocatalytic cycle, in particular for the fuel generating water splitting reaction, requires a reductionist approach to address separately the effect of size, shape and composition of each component towards the rational design of an efficient photocatalytic system.

The role of the exciton dynamics, related to the semiconductor component, has been investigated in particular in prototypical TiO_2_-based systems and in colloidal semiconductor-metal hybrid nanorods (NRs). These studies pointed out to the potential of tailoring the semiconductor component in the hybrid nanoparticles, permitting fine tuning of the light absorption and electron–hole dissociation as preliminary steps to charge separation and catalysis^9^,^10^,^11^. The metal co-catalyst component has a particularly important role in the photocatalytic cycle. The charge separation from the semiconductor is directly affected by the metal domain characteristics and the actual catalysis occurs on its surface^12^,^13^. Control over the size of the metal domain and its optimization is an essential parameter for the rational photocatalytic system design. The size dependence of catalysis on bare Au islands deposited on titania was investigated revealing sharp optimal catalytic performance for CO oxidation at island thickness of ∼2 atomic layers corresponding to ∼3 nm in diameter^14^, and in the seminal work of Goodman and co-workers, this was attributed to a metal to non-metal transition^15^. In relation to photocatalytic activity, early studies discussed the effect of size on Fermi-level equilibration related to charging of the metal domain following irradiation of the system^16^,^17^. The effect of the metal co-catalyst size, particularly on the hydrogen generation, was addressed in the context of TiO_2_–Au and CdS-Pt with multiple metal domains^18^,^19^. While the former observed no size effect for the Au domain varying between 3 and 12 nm, the latter has reported optimal hydrogen generation in extremely small Pt clusters (∼50 atoms). However, a detailed mechanistic description, particularly in view of these diverse behaviours, is missing and is important for further development of such hybrid nanoparticles in the context of photocatalysis.

In this work we address the effect of the metal co-catalyst size on the photocatalysis process, using hybrid semiconductor-metal nanoparticles with a single catalytic domain as a model system. By a combination of ultrafast transient absorption measurements, hydrogen generation yield study and theoretical modelling, we observe and explain a non-monotonic metal domain size dependence of the hydrogen generation efficiency.

## Results

### Size-controlled Au tip growth on CdS NRs

**Figure 1:**
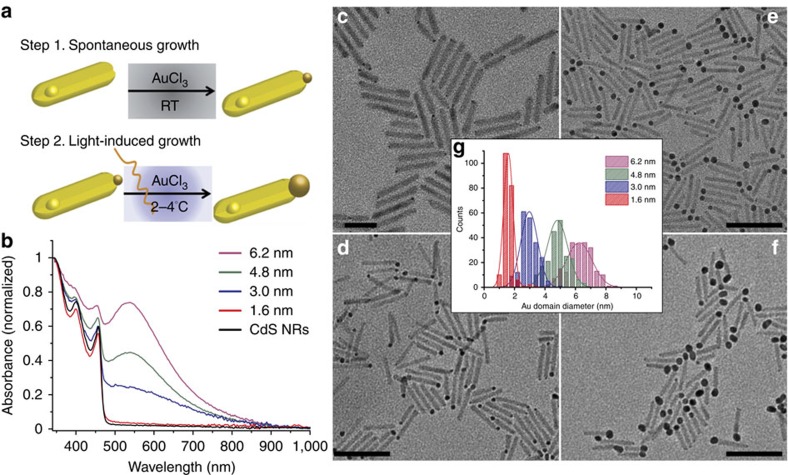
Growth of size-controlled semiconductor-metal nanohybrids. (**a**) A scheme of the two-step metal growth deposition. (**b**) UV-vis absorbance spectra of CdS-Au hybrid nanoparticles showing the development of the plasmonic feature ∼540 nm as the Au tip size increases. TEM images of CdS-Au hybrid nanoparticles with 1.5±0.2 nm Au tip size after 1 h dark synthesis (**c**) and light-induced synthesis for 30 min with various CdS:Au molar ratio leading to Au tip size of of 3.0±0.5 nm (**d**) 4.8±0.7 nm (**e**), and 6.2±0.8 nm (**f**). Scale bars; **c**, 20 nm; **d**–**f**, 50 nm. (**g**) size distribution histogram of the Au metal tip diameters.

CdS NRs of 31.6 nm length and 3.9 nm diameter were synthesized using modification of a previously reported procedure employing seeded growth^20^ (Supplementary Fig. 1). Site-selective Au deposition on a single-rod apex, with high control of the metal tip size, was achieved by combining two different synthetic approaches consecutively (Fig. 1a). First, a dark reaction was used to obtain site-selective small metal islands growth on the apexes of the CdS NRs (refs 21, 22). As can be seen in the transmission electron microscopy (TEM) image in Fig. 1c, a single small metal-tip of ∼1.5–1.8 nm diameter grows on one apex with narrow size distribution (∼7%). This selective growth takes place due to the favoured surface reactivity of the (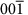) facet of the CdS rod, that encourages the heterogeneous nucleation of gold. Furthermore, the use of long alkyl chain amines such as octadecylamine, instead of shorter ones, minimizes Au nucleation on the less reactive facets such as the sides of the rod. Owing to the length of the alkyl chain a phase transition to a static phase occurs at lower temperatures thus blocking the Au precursor's access to the NR surfaces^23^. These small Au domains serve as seeds for the second step, using light-induced Au growth under inert atmosphere and at low temperature, 2–4 °C (Supplementary Methods). This approach allows for size control by changing the irradiation times and the Au^3+^/NRs ratio. Figure 1d–f show TEM images of CdS-Au hybrid nanoparticles with different Au tip sizes and with narrow size distribution (Fig. 1g). Figure 1b presents the absorption spectra of the bare CdS NRs and CdS-Au hybrid nanoparticles with different Au tip sizes. All spectra exhibit a similar sharp rise at 460 nm related to the onset of the CdS NRs absorption. Several absorption features are seen to the blue of the onset, related to the band gap and to higher excited optical transitions of the CdS NRs, which signify the good size monodispersity of the sample. A plasmon peak develops at 540 nm, correlated with the increase of the metal tip size. Phase transfer to aqueous solution was performed with polyethyleneimine which was recently reported as high performance surface coating for photocatalytic applications and provides good colloidal stability^11^.

### Transient absorption measurements of charge separation

**Figure 2:**
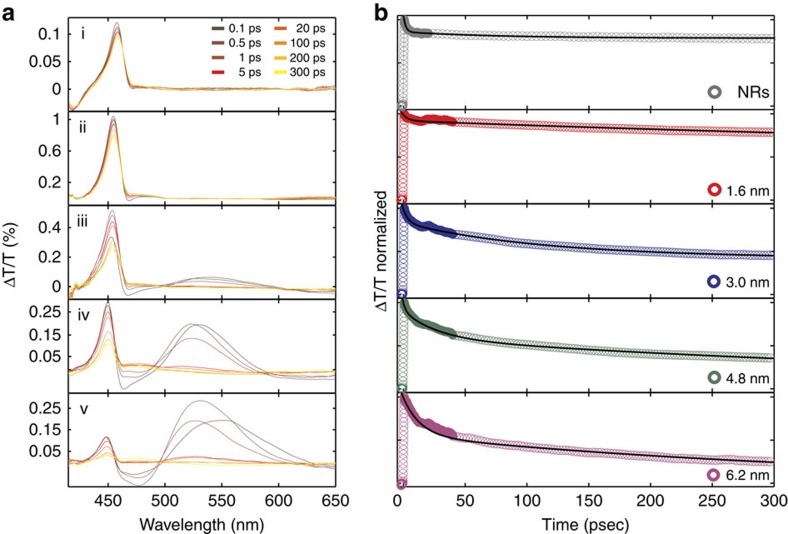
Ultrafast spectroscopy of nanohybrids. (**a**) Transient absorption spectra of CdS NRs (i) and CdS-Au hybrid nanoparticles for different Au metal tip sizes including 1.6 nm (ii), 3.0 nm (iii), 4.8 nm (iv) and 6.2 nm (v) at 450 nm excitation. (**b**) Corresponding normalized transient absorption dynamics of the bleach recovery at 450 nm, attributed to the first excitonic transition of the CdS NR component for CdS NRs and CdS-Au hybrid nanoparticles with different Au metal tip sizes.

The effect of the different Au domain sizes on charge carrier dynamics was studied using broadband ultrafast transient absorption spectroscopy. In Fig. 2a we show the time-resolved differential transmission (ΔT/T) spectra for the CdS NRs and the series of Au tip sizes. Following 450 nm optical excitation and formation of excited electron–hole pairs, the ΔT/T spectra at early delay times reveal a pronounced bleach around 450 nm in both CdS NRs and CdS-Au hybrid NRs attributed to depletion of the first excitonic transition in the CdS rods due to electron state filling^24^. In addition, for the larger Au tips, a broad bleach feature develops ∼540 nm, which corresponds to the plasmonic feature of the Au tips^25^. The decay of the plasmonic feature, by electron–phonon scattering, showed no size dependence and the measured lifetime for all Au domain sizes was 1.5–1.7 ps (Supplementary Figs 6–8) consistent with prior reports for colloidal Au nanoparticles^25^,^26^.

Comparison between the normalized transient absorption dynamics for the bleach recovery in the spectral region of the CdS band gap exciton is presented in Fig. 2b for the different Au tip sizes. In the bare CdS NRs (upper panel, Fig. 2b), a fast decay component with a small amplitude is observed, and is related to residual cooling of the electrons to the CdS conduction band edge. This is followed by a long decay component, corresponding to exciton recovery to the ground state. These bleach recovery kinetics were fitted to a bi-exponential function and resulted in a time constant for electron–hole recombination of 3.4 ns (Supplementary Table 3). At the presence of a metal tip a clear additional timescale is introduced, slower than the rapid cooling and faster than the electron–hole recombination. The amplitude of the intermediate decay process increases with increasing metal domain size. We assign this additional timescale to the electron transfer from the CdS to the metal tip. The measured kinetic decays were fitted to a tri-exponential function (Supplementary Note 3). The charge transfer rates extracted from this fit were found to be 16 ps for 6.2 nm, 29 ps for 4.8 nm, 103 ps for 3.0 nm and 770 ps for 1.6 nm Au tips sizes, with amplitudes decreasing from 40 to 15% for smaller tip sizes.

### Photocatalytic hydrogen production efficiency measurements

**Figure 3:**
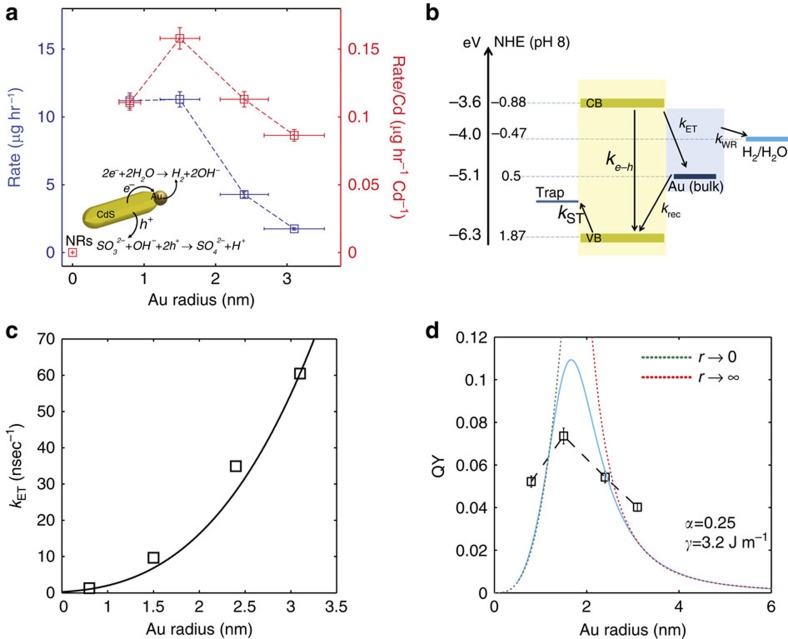
Understanding size-dependent hydrogen production yield. (**a**) Hydrogen production rate (blue) and Cd normalized rate (red) curves as a function of Au size domain in the hybrid nanoparticles. Negligible rates are measured for the CdS NRs. (**b**) Energy band alignment diagram with relevant photocatalytic processes kinetic routes. (**c**) Measured semiconductor-metal electron transfer (*k*_ET_) rates (squares) and fitting modified Fermi golden rule model for this process (solid line). (**d**) Measured QY (black squares connected by dashed line) along with the non-monotonic kinetic model behaviour (blue solid line). Green and red dotted lines present limiting behaviours of the model for zero and infinite metal domain sizes, respectively. Error bars in **a** and **d** indicate the Au tip size distribution and the uncertainty in the hydrogen production rate.

The photocatalytic activity of the same hybrid nanoparticles was measured via the light-induced reduction of water at 405 nm to produce hydrogen in the presence of Na_2_S-9H_2_O and Na_2_SO_3_, acting as sacrificial hole scavengers as depicted schematically in Fig. 3a (inset)^27^. The amount of hydrogen gas produced was determined using a gas chromatograph equipped with a thermal conductivity detector. Figure 3a, blue curve, shows the hydrogen generation rate versus Au tip size. A weak dependence, observed for the two smallest sizes, is followed by a marked decrease of the rate in the larger tips, overall by a factor of nearly 10 comparing the maximum and the minimum rates.

These results represent the actual hydrogen evolution rates, but they do not account for photons that are absorbed directly by the intraband transitions of the metal tip and do not contribute to the generation of hydrogen due to their rapid relaxation. The total absorption of such CdS-Au hybrid nanoparticles can be considered in first approximation as the superposition of the contributions of the exciton and the plasmon^28^. The red curve in Fig. 3a corrects for this, by normalizing the rates to the semiconductor component absorption. The actual concentration of each sample was obtained by inductively coupled plasma mass spectrometry (ICP-MS) analysis to determine the Cd content. Therefore the red curve is normalized to the overall Cd content, which is proportional to the contribution of the semiconductor to the nanohybrid absorption. This normalization is therefore expressing more cleanly the essential metal domain size effect in hydrogen reduction. It reveals a non-monotonic dependence in which an intermediate Au tip size provides the optimal hydrogen evolution rate for the photocatalytic water reduction and the highest value for the hydrogen evolution quantum yield (QY).

Additional aspect of hydrogen generation rate normalization can be considered to isolate the Au metal domain effect on the efficiency of this photocatalytic process. This may be considered by normalizing the hydrogen production rate to the loading of the co-catalyst component^18^. X-ray photoelectron spectroscopy measurements on the hybrid nanoparticles (HNPs) with different Au metal domain sizes show a dominant doublet at the Au 4*f* binding energy (BE) consistent with the zero-valent Au peak (84.0 eV), which designates the metallic nature of these domains. Intensities of the Au 4*f*_*7/2*_ and 4*f*_*5/2*_ peaks show a pronounced increase in the intensity correlated to the larger metal domain sizes (Supplementary Fig. 4). Quantification of the atomic concentration percent of each element in the HNPs structure presented in Supplementary Table 2 allows the calculation of the Cd:Au ratio in each HNPs sample. The nearly 10-fold increase of this relative atomic concentration of the Au between the different metal domain sizes indicates the significantly higher loading of the Au metal on the CdS semiconductor NR structure with increased Au tip size, while the minor change in the Cd or S atomic concentration implies lack of substantial change in the rod dimensions. Consequently, normalization of the hydrogen production rate by the Cd:Au ratio is considered as normalization to the Au metal loading. The results of such correction for the measured hydrogen generation rates are fully consistent with the CdS absorption normalization obtained by ICP-MS discussed above, and reveal a similar non-monotonic behaviour as function of Au metal domain size (Supplementary Fig. 5 and Note 2).

## Discussion

To rationalize the size dependence of the non-monotonic photocatalytic behaviour along with the charge transfer dynamics we propose a minimalistic model consisting of several kinetic steps, as sketched in Fig. 3b and Supplementary Fig. 9. The photo-excited electron in the semiconductor can relax by electron transfer to the co-catalyst metal domain (with a rate given by *k*_ET_) or by electron–hole recombination (with rate *k*_*e–h*_). The latter is rather slow^1^ compared with the other processes and is assumed to be constant independent of the Au tip size (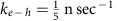). The electron on the metal tip can either promote water reduction reaction (*k*_WR_) or undergo back recombination with the hole that is left behind on the semiconductor domain (*k*_rec_), which is modelled by the hole transfer from the semiconductor to the metal tip. An additional important process is the trapping of the hole (*k*_ST_) with a rate that is comparable to that of electron transfer into the metal tip^29^. Once the hole is trapped it blocks the electron transfer channel by the electron*–*hole Coulomb interaction, which also leads to a localization of the electron^11^,^30^.

The efficiency of hydrogen production can be obtained in a closed form and is given by the *t*→∞ of the solution to the master equation (Supplementary Note 4):


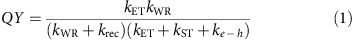


to make contact with the measured hydrogen production efficiency the different rates appearing in equation (1) need to be determined. In fact, some of these rates can be determined independently by combining the transient absorption measurements with a suitable theoretical model. The semiconductor-metal electron transfer (*k*_ET_) and the back recombination (*k*_rec_) rates can be described by Auger processes with values given by Fermi's golden rule:


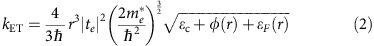


and


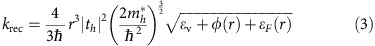


where *r* is the metal domain radius, 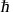 is the Planck's constant divided by 2*π*, 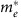 and 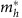 are the effective mass of the electron and the hole in the metal, *t*_*e*_ and *t*_*h*_ are the electron and hole tunnelling matrix elements (assumed to be independent of *r*), *ɛ*_c_*, ɛ*_v_, 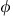
(*r*) and *ɛ*_F_(*r*) are the semiconductor conduction and valance band energies, the metal work-function and the metal Fermi-level measured from the bottom of the energy band. Note that both rates depend on the metal tip radius *r* through the steep dependence of the density of states on the tip volume (*r*^3^), and weakly through the dependence of the Fermi energy^31^ (*ɛ*_F_(*r*)) and work function^32^,^33^ on *r*


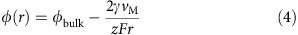


where 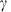 is the surface tension, 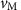 is the molar volume, 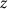 is number of transferred electrons, 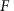 is Faraday's constant.

The water reduction reaction on the metal co-catyalyst is described by the cathodic rate in the Butler–Volmer model^34^ for redox reactions. The electron transfer process is given by a Marcus-like expression^35^:


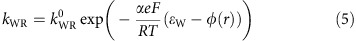


where *k*_WR_ is the electron reduction rate of an absorbed water molecule, 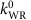 is the standard rate constant, *R* is the gas constant, *T* is the temperature, *α* is the electron transfer coefficient which determines the symmetry of the transition state (for example, 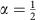 corresponds to a transition state with equal contributions from the reactants and products), and *ɛ*_W_ is the water reduction potential. The anodic rate for the hydrogen oxidation (back reaction) can be neglected because the hydrogen concentration is small compared with the proton concentration at the experimental pH level. In contrast to the electron transfer and recombination rates given by equations (2) and (3), *k*_WR_ depends exponentially on size of the metal domain through the dependence of 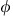
(*r*).

We now have a working model to analyze the governing factors in the metal domain size effect on electron transfer and photocatalytic efficiency, and compare them with the experimental results. First, in Fig. 3c we determine *k*_ET_ by fitting the measured electron transfer rates obtained from the transient absorption to equation (2). The overall size dependence of *k*_ET_ is in good agreement with the theoretical *r*^3^ prediction. The only fitting parameter used is the tunnelling matrix element, *t*_*e*_=5.6 × 10^−5^ eV. This seemingly small value is consistent with the results of Kamat on semiconductor-metal oxide hybrid nanoparticles^36^. The remaining parameters were taken from the literature and are provided in Supplementary Note 4.

Next, we calculate the efficiency of hydrogen production and compare the prediction to the experimental values, as shown in Fig. 3d. To capture the essential non-monotonic behaviour observed experimentally, we studied the dependence of the QY on the various parameters as shown in detail in the Supplementary Figs 10–12. In particular, the dependence on the hole tunnelling matrix element, *t*_*h*_ , was studied (Supplementary Fig. 10) and a reasonable description of the measured efficiencies is obtained for *t*_*h*_=1.1 × 10^−6^ eV, up to 50-folds smaller than *t*_*e*_ to yield meaningful results. This low value corresponds to an electron residence time on the Au tip sufficiently long to allow for effective water reduction. Indeed, the back recombination rate under these parameters for the different metal sizes is in the range of sub-μsec to several μsec for large to small Au domain sizes respectively, consistent with experimental results for Pt tips on CdS rods^1^.

Two other parameters are needed for the qualitative description of the experimental results by the model. For the metal surface tension, *γ*, several reported values for gold nanoparticles were tested (Supplementary Fig. 11)^32^,^37^,^38^. Reasonable surface tension values (*γ*) are between 2.5 and 4 J m^−1^. Higher surface tension values result in high work function values for the metal domain above the semiconductor conduction band minimum leading to vanishing electron injection rates. Furthermore, in larger tip sizes the efficiency is overestimated due to large overpotential relative to the standard water reduction potential at the experimental pH. Finally, for the electron transfer coefficient, *α*, the fits yield values around *α*=0.25 (Supplementary Fig. 12), in the range of values reported in the literature for similar systems^34^,^39^. This implies that the transition state for the water reduction is closer to the reactants.

A particular kinetic model solution is presented in Fig. 3d (solid blue curve) and manifests the non-monotonic dependence of the QY on the Au tip size consistent with the normalized experimental QY (squares connected by dashed line). The non-monotonic dependence arises from the opposing behaviour of the QY in the limits of *r*→0 and *r*→∞. For the former, we find that QY∝*r*^3^ increases with the tip radius, as the rate determining step is electron injection into the metal tip. For the latter, 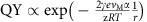 decreases exponentially with the tip radius where the rate determining step is the reduction of water as the back hole transfer competes with the reduction. Hence, an intermediate Au tip size provides an optimum balancing between the charge separation rate and efficiency, and the back recombination competing with the water reduction.

In conclusion, an optimal metal domain size is found for photocatalytic water reduction using hybrid semiconductor-metal NRs. The optimal value is explained in terms of competing processes where for small tips the hydrogen evolution QY is mainly determined by the rate of electron injection to the metal tip, whereas for large tips it is determined mainly by the water reduction on the metal surface. These two limits show opposite dependence on the metal domain size, leading to an optimal value as explained by a minimalist and general kinetic model with parameters fitted to reproduce qualitatively the experimental results, in particular after normalizing out the metal domain absorption effects. Thus, the behaviour is general and not limited to the metal type or the reduction reaction, and can be used for rational design of photocatalysts based on hybrid semiconductor-metal nanostructures.

## Methods

### Materials and syntheses

All chemical precursors used for this study were purchased from Sigma Aldrich, PCI Synthesis, and Merck. See Supplementary Methods for more details.

Synthesis and phase transfer of CdS NRs and CdS-Au hybrid nanoparticles are described in details in Supplementary Figs 1,2 and Supplementary Methods.

### Nanoparticle characterization

TEM and high resolution scanning transmission electron microscope characterization was performed using a Tecnai T12 G2 Spirit and Tecnai F20 G2, respectively. All size statistics are done with ‘Scion image' programme on 200 particles. Absorption was measured with a JASCO V-570 UV-vis-near IR spectrophotometer. Extinction coefficient values of the nanoparticles were calculated using a previously reported method^40^.

### Inductively coupled plasma mass spectrometry measurements

Following hydrogen generation kinetic measurements a 100 ml of HNPs solution was etched overnight in 1 ml of 69% nitric acid. Following sonication, 100 μl of the HNPs solution was mixed with 3.35 ml of three distilled water and analysed by ICP-MS (c × 7500, Agilent) for Cd. The quantity of Cd in each solution was calculated using external calibration with standard Cd solutions (Supplementary Table 1).

### Hydrogen evolution rate and efficiency measurements

To determine and measure the evolved hydrogen gas from the photocatalytic reaction using the HNP model systems, the following set-up is used. The photocatalysts were dispersed in three distilled water solution (2 ml; optical density, OD ∼1 at 405 nm). The photocatalyst solution was placed in a quartz cuvette and hole scavengers, Na_2_S-9H_2_O and Na_2_SO_3_, (typically 0.05 and 0.07 M, respectively), were added to the water. The solution is purged with argon for 20 min and stirred. The hybrid nanoparticles were then illuminated with 40 mW 405 nm laser. Aliquots of the reaction vessel head space were taken using a gas tight syringe at different time intervals and detected and quantified using Varian gas chromatograph (model 6820) equipped with a molecular sieve (5 Å) packed column and a thermal conductivity detector. The resulting chromatograms and hydrogen concentration are obtained by the comparison to a calibration curve of known hydrogen amounts (Supplementary Fig. 3 and Note 1).

### Ultrafast transient absorption measurements

The laser system employed for ultrafast transient absorption was based on a Ti-Sapphire chirped pulse amplified source, with maximum output energy of ∼800 μJ, 1 kHz repetition rate, central wavelength of 800 nm and pulse duration of about 150 fs. Excitation pulses at 400 nm were obtained by doubling the fundamental frequency in a β-barium borate crystal while other pump photons at different wavelength were generated by non-collinear optical parametric amplification in β-barium borate, with pulse duration ∼100 fs. Pump pulses were focused to 175 μm diameter spot. Probing was achieved in the visible range by using white light generated in a thin sapphire plate, and in the UV-visible range by using a thin Calcium Fluoride plate. Chirp-free transient transmission spectra were collected by using a fast optical multichannel analyser with dechirping algorithm. The measured quantity is the normalized transmission change, ΔT/T.

## Additional information

**How to cite this article:** Ben-Shahar, Y. *et al.* Optimal metal domain size for photocatalysis with hybrid semiconductor-metal nanorods. *Nat. Commun.* 7:10413 doi: 10.1038/ncomms10413 (2016).

## Supplementary Material

Supplementary Information Supplementary Figures 1-12, Supplementary Tables 1-3, Supplementary Notes 1-4, Supplementary Methods and Supplementary References.
