# Supplementary material for: Optimal metal domain size for photocatalysis with hybrid semiconductor-metal nanorods
Source: Nat Commun. 2016 Jan 19;7:10413. doi: 10.1038/ncomms10413 (PMC4735686; doi:10.1038/ncomms10413)
Supplement: Supplementary Information — Supplementary Figures 1-12, Supplementary Tables 1-3, Supplementary Notes 1-4, Supplementary Methods and Supplementary References. [file ncomms10413-s1.pdf]

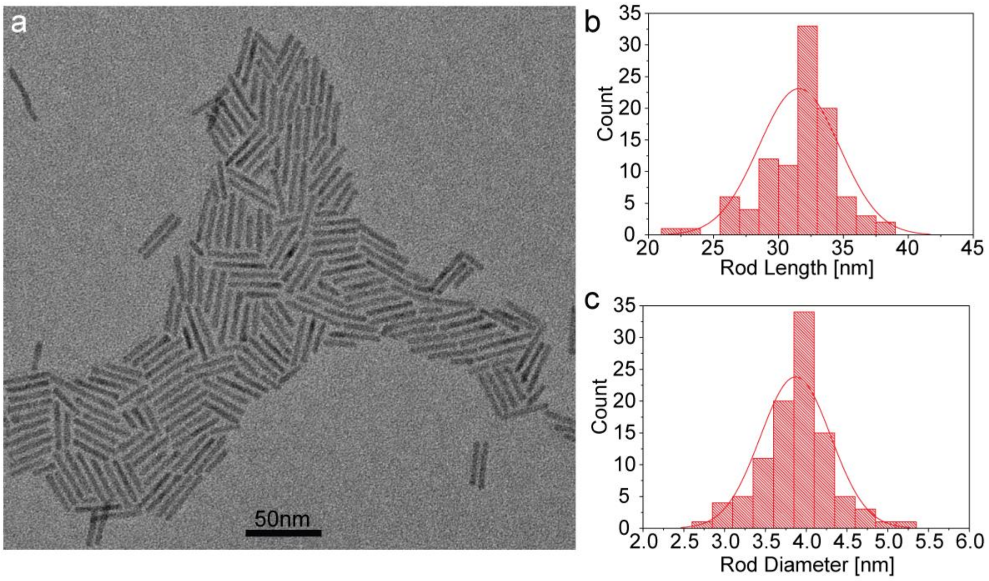

Supplementary Figure 1. a, TEM image of CdS NRs (31.6x3.9 nm). Sizing histograms for **b,** CdS NRs length and **c,** CdS NRs diameter.

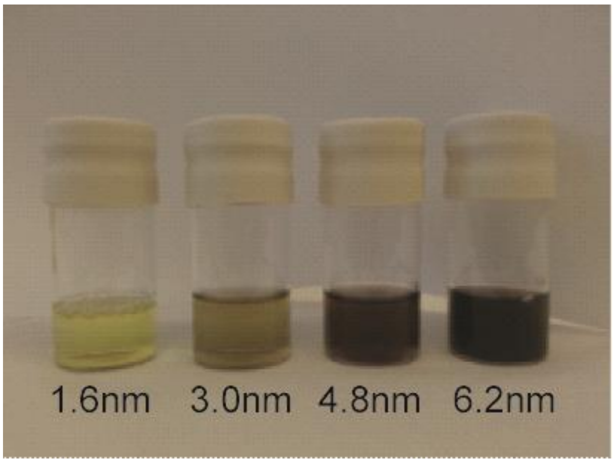

Supplementary Figure 2. CdS-Au HNPs with different metal domain diameter sizes coated by PEI polymer in aqueous solution.

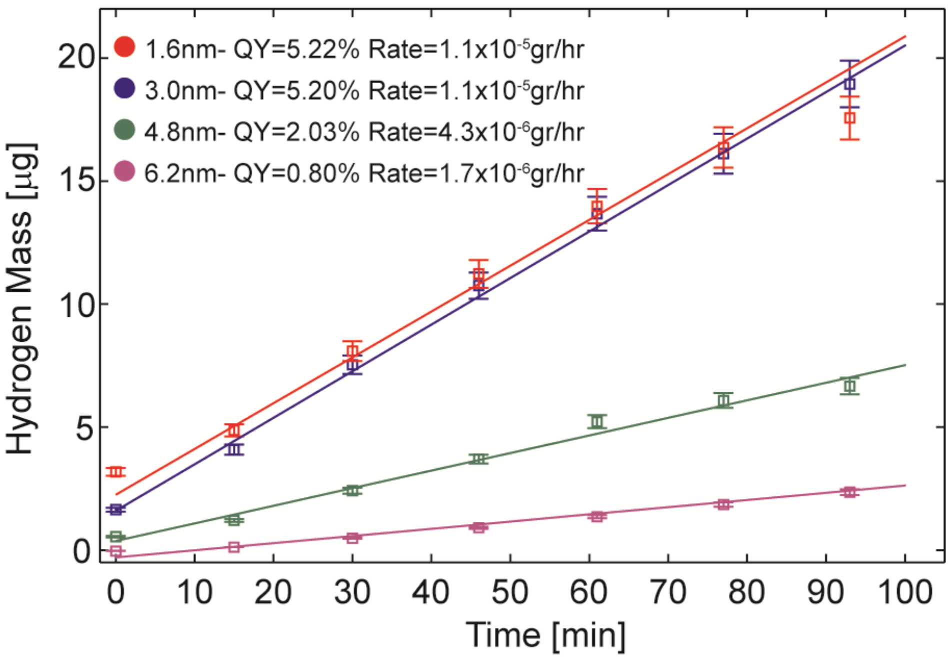

Supplementary Figure 3. Kinetic measurements of hydrogen production and calculated apparent quantum yield by different metal domain sizes of CdS-Au HNPs (PEI coated) in presence of Na_2S-$9H_2O$ and Na_2SO_3 as hole scavengers.

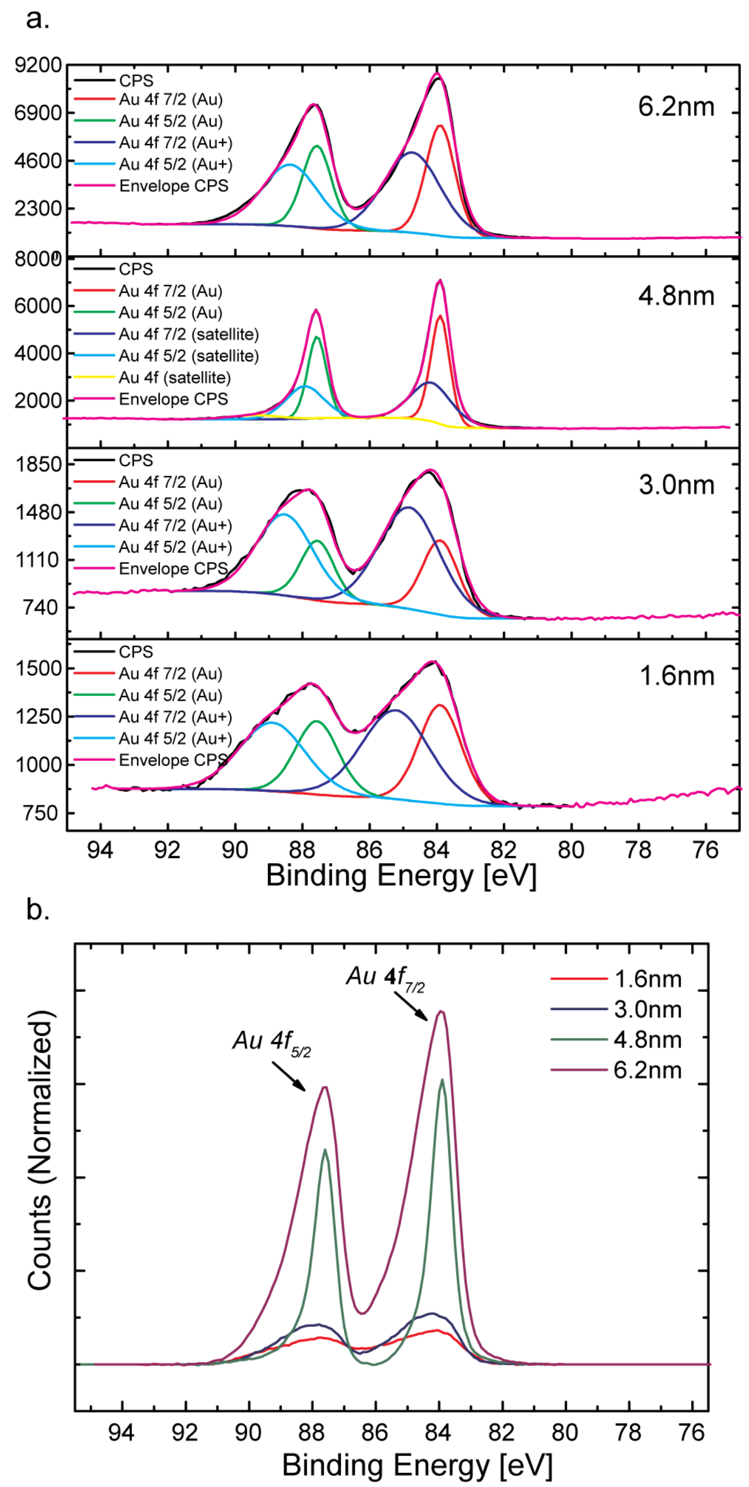

Supplementary Figure 4. **a,** High resolution X-ray Photoelectron Spectroscopy (XPS) spectra of Au $4f_{7/2}$ and $4f_{5/2}$ of HNPs with different Au metal domain sizes, along with peak fitting. **b,** normalized Au peak intensities for HNPs with different Au metal domain sizes.

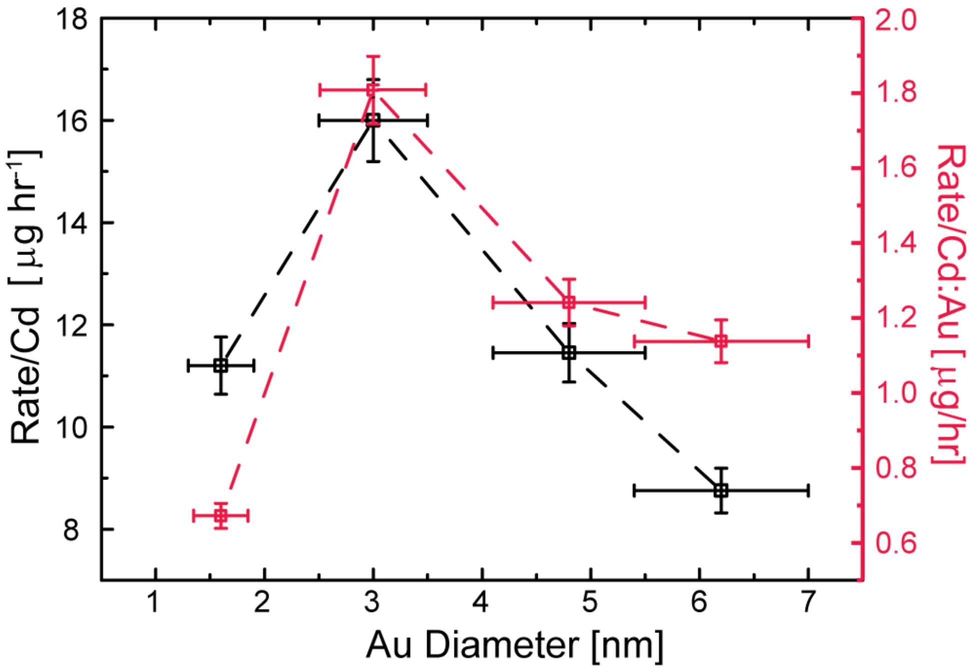

Supplementary Figure 5. Hydrogen production rate normalized by CdS semiconductor component absorption (black curve) and by the Cd:Au ratio (red curve) quantified via XPS measurements as a function of Au size domain in the hybrid nanoparticles.

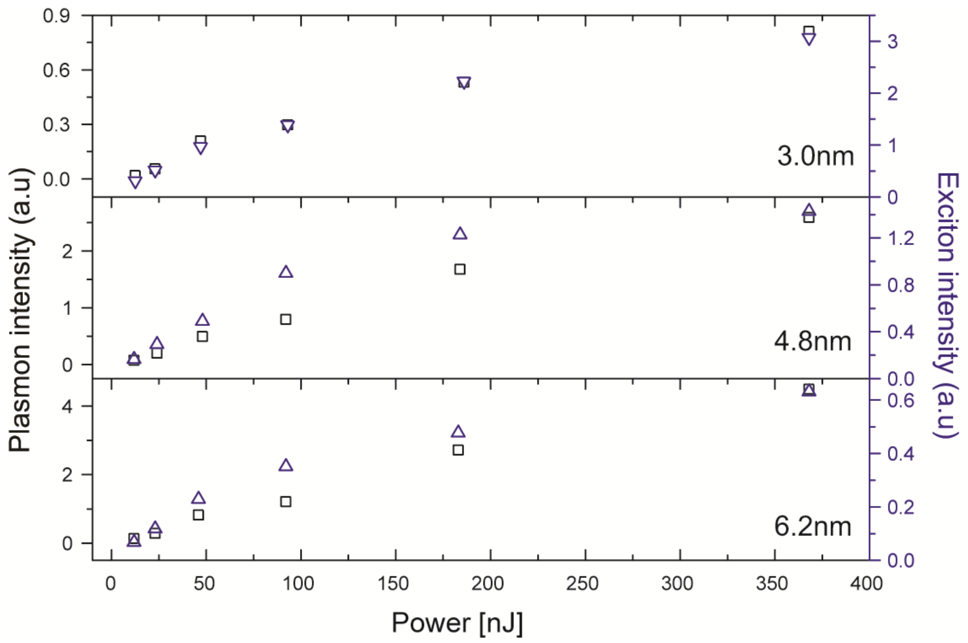

Supplementary Figure 6. Power dependent TA measurements of different metal domain sizes of CdS-Au HNPs pumped at 450nm. Plasmon (black square) and exciton (blue triangle) intensities probed at 540nm and 450nm, respectively, versus light source excitation power.

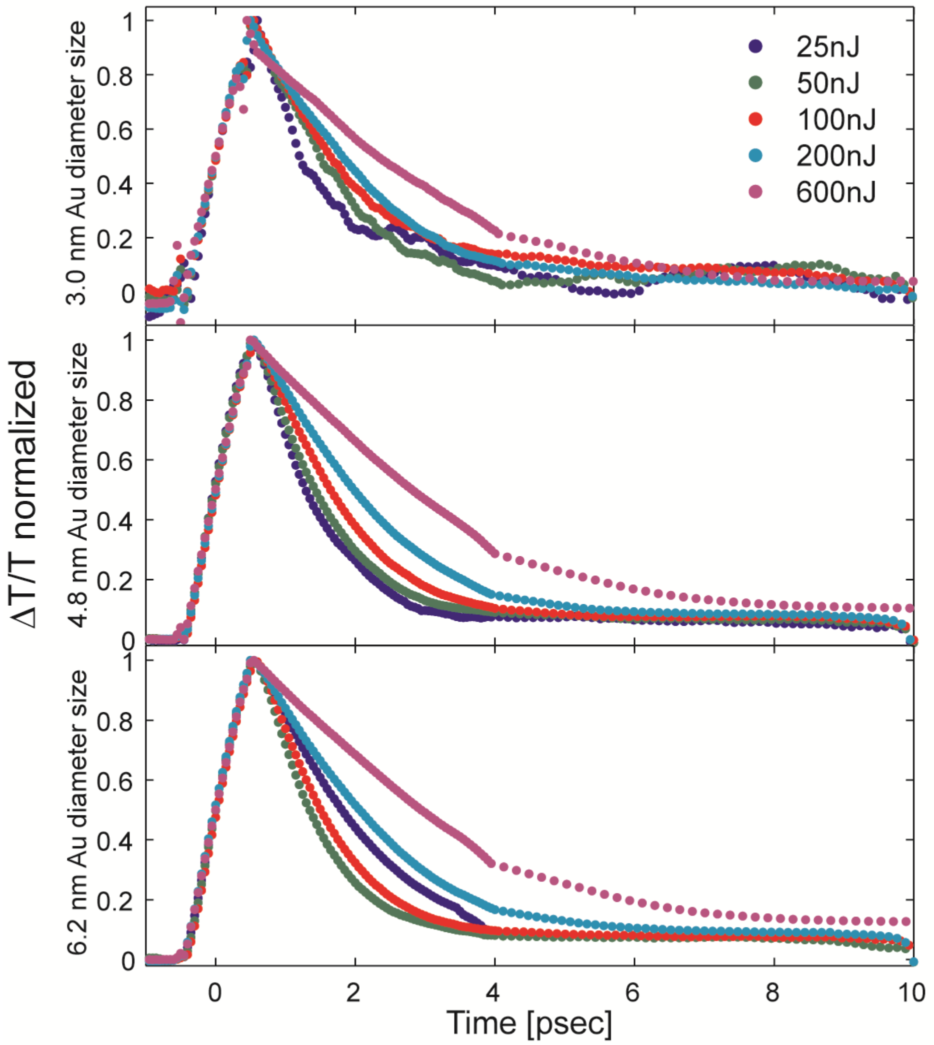

Supplementary Figure 7. Power dependent TA measurements of different metal domain sizes of CdS-Au HNPs pumped at 590nm and probed at 540nm with various excitation powers.

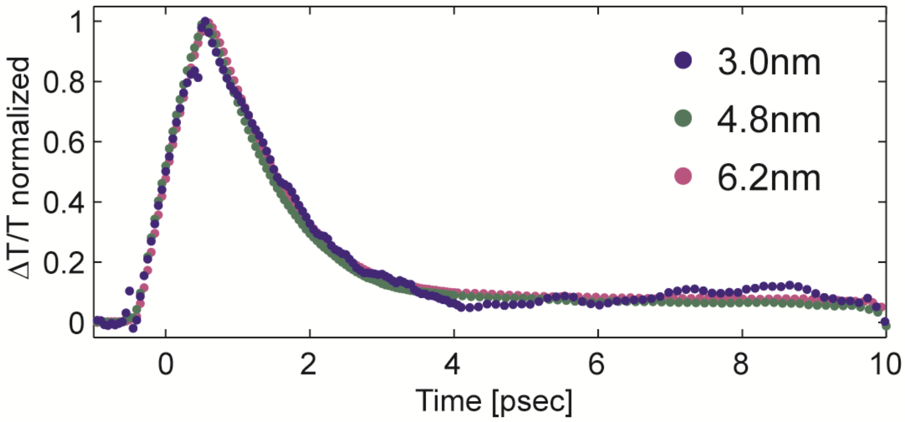

Supplementary Figure 8. Plasmon dynamics at 590nm and 50nJ pump excitation properties of different metal domain sizes of CdS-Au HNPs probed at 540nm.

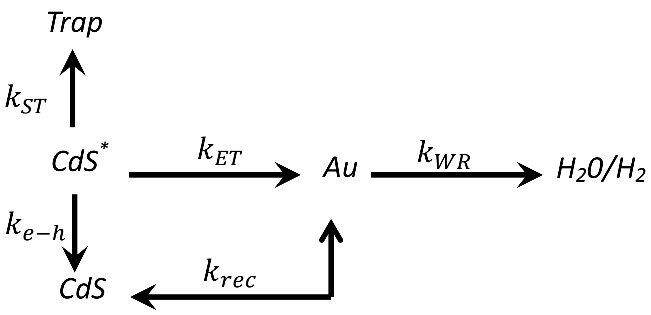

Supplementary Figure 9. Kinetic scheme of photocatalytic activity of the hybrid nanoparticles**.** k_{ET} is the electron transfer rate from the CdS excited state to the Au metal domain. k_{rec} is the electron transfer rate from the Au metal domain to the ground state of CdS NRs. (This equals the hole transfer rate to the metal). k_{WR} is the electron reduction rate of water molecule at the surface of the Au domain. k_{ST} is the electron or hole trapping rate. k_{e-h} is the recombination rate to the ground state of CdS valance band.

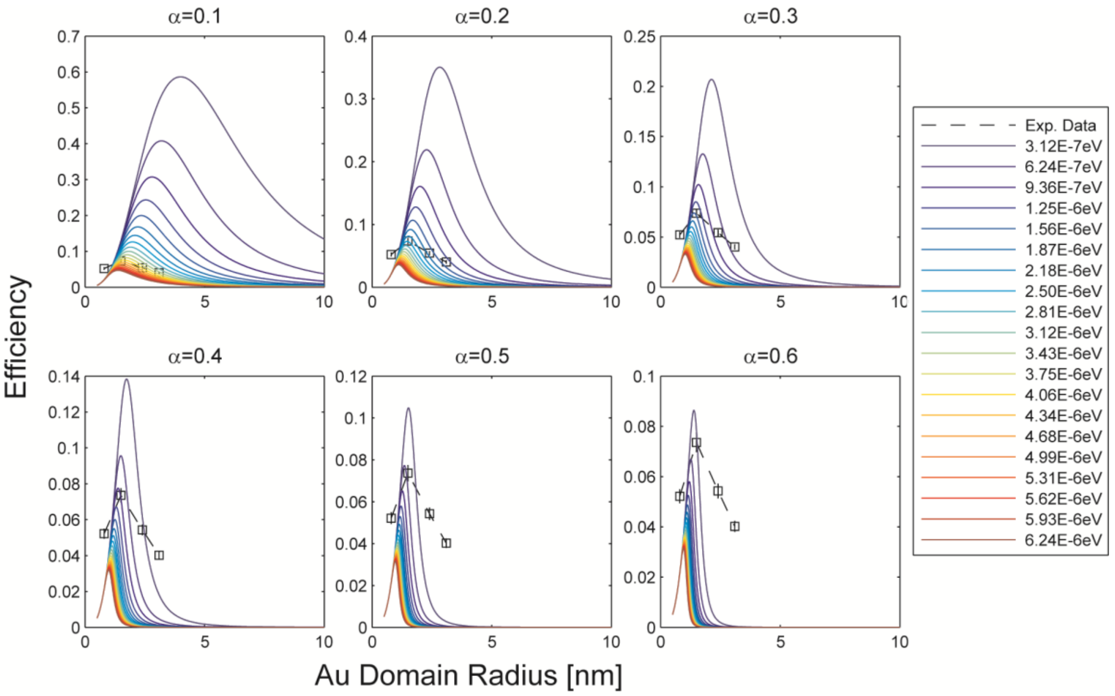

Supplementary Figure 10. Modeled hydrogen generation efficiency dependence on the hole tunneling matrix element at symmetry factor between 0.1-0.6 along with experimental hydrogen generation efficiencies for different metal domain radius sizes. While keeping other parameter values constant γ=3.19 J m^{-1}, $t_e = 5.6 \times 10^{-5}$ eV, k_{ST}=1/18 psec^{-1}, and k_{e-h}=1/5 nsec^{-1}.

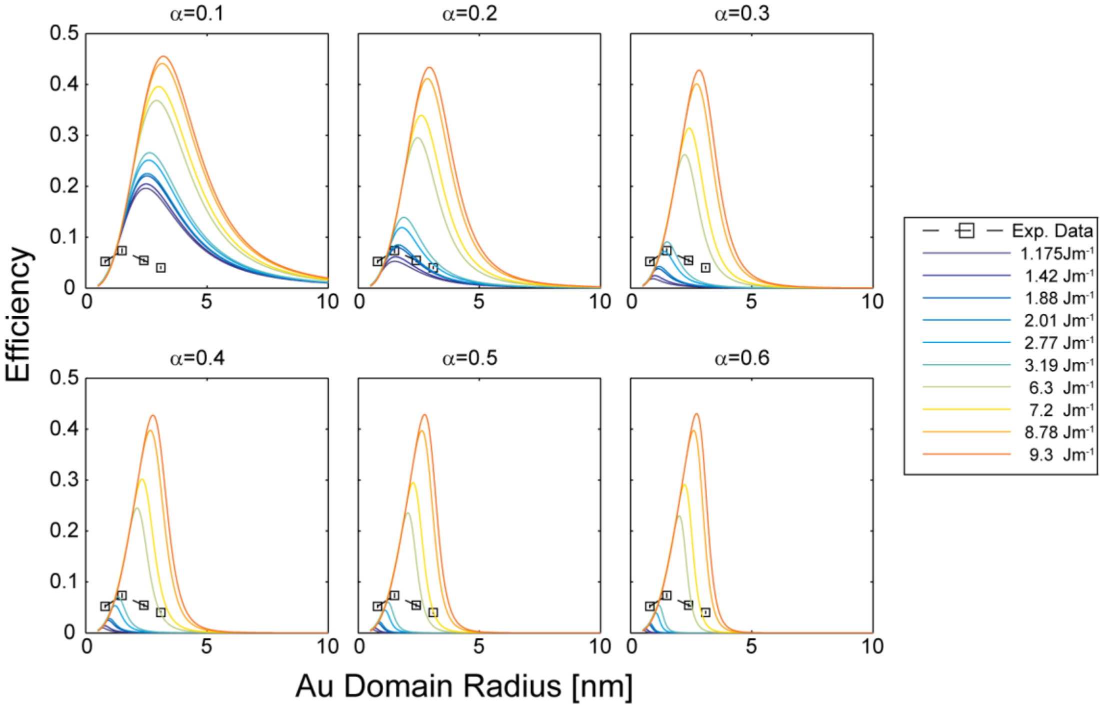

Supplementary Figure 11. Modeled hydrogen generation efficiency dependence on the surface tension at symmetry factor between 0.1-0.6 along with experimental hydrogen generation efficiencies for different metal domain radius sizes. While keeping other parameter values constant $t_e = 5.6 \times 10^{-5}$ eV, $t_h = 1.1 \times 10^{-6}$eV, k_{ST}=1/18 psec^{-1}, and k_{e-h}=1/5 nsec^{-1}.

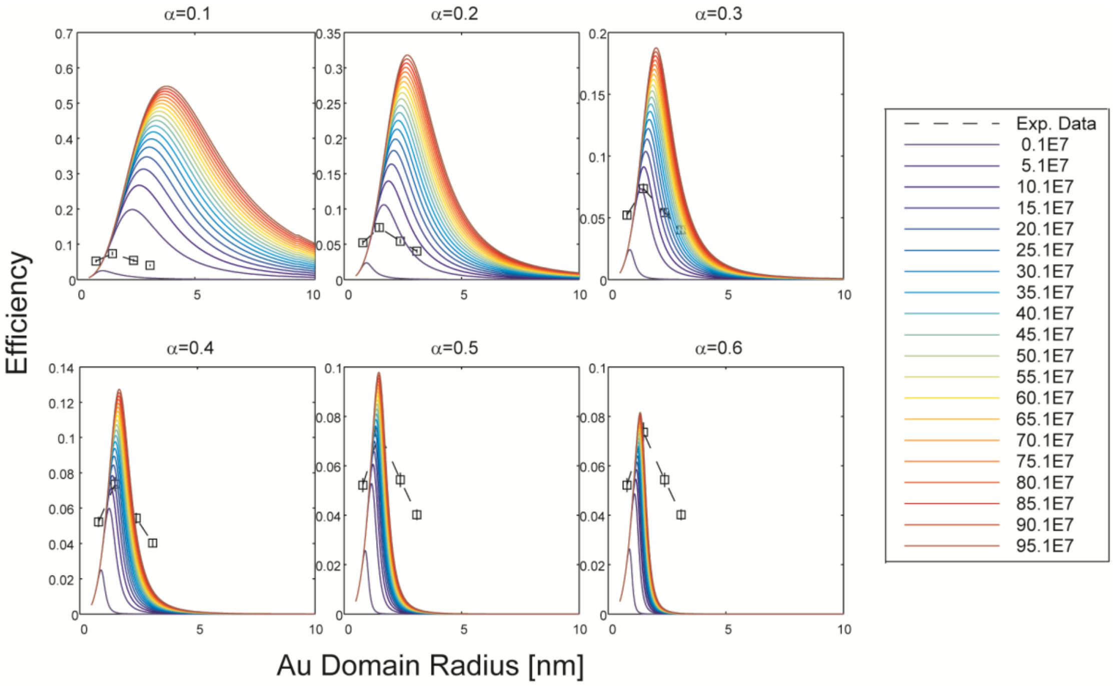

Supplementary Figure 12. Modeled hydrogen generation efficiency dependence on the conditional rate constant at symmetry factor between 0.1-0.6 along with experimental hydrogen generation efficiencies for different metal domain radius sizes. While keeping other parameter values constant $t_e = 5.6 \times 10^{-5}$ eV, $t_h = 1.1 \times 10^{-6}$eV, k_{ST}=1/18 psec^{-1}, and k_{e-h}=1/5 nsec^{-1}.

Supplementary Tables

Supplementary Table 1. Cd^{+} ion concentrations of the different HNPs samples obtained by ICP-MS (diluted 33 times)

	No Au	1.6nm	3.0nm	4.8nm	6.2nm
**Avg. Cd [ppb]**	196.9 (073%)	251.9 (1.21%)	177.9 (0.81%)	94.2 (1.18%)	50.4 (1.06%)

Supplementary Table 2. Quantification of atomic concentration percent and Cd:Au ratio for HNPs with different Au metal domain sizes.

	**Atomic Concentration %**			
	**1.6nm**	**3.0nm**	**4.8nm**	**6.2nm**
**Cd 3*d***	57.74	53.27	49.11	40.41
**S 2*p***	38.75	38.25	36.53	33.14
**Au 4*f***	3.51	8.48	14.36	26.45
	**Atomic Concentration Ratio**			
**Cd:Au**	16.66	6.25	3.45	1.54

Supplementary Table 3. Fitting parameters for HNPs tri-exponential and NRs bi-exponential fitting:

$$Y(x) = A * exp(-(1/\tau_1) * x) + B * exp(-(1/\tau_2) * x) + C * exp(-(1/\tau_3) * x)$$

$$Y(x) = A * \exp\left(-\left(\frac{1}{\tau_1}\right) * x\right) + B * \exp\left(-\left(\frac{1}{\tau_2}\right) * x\right)$$

	A	τ_1(ps)	B	τ_2(ps)	C	τ_3(ps)
**NRs**	0.15	2.8	0.85	3378	-	-
**1.6nm**	0.07	4.4	0.15	767	0.76	2500
**3.0nm**	0.16	4.9	0.29	103.4	0.53	1400
**4.8nm**	0.10	2.3	0.32	28.64	0.58	595
**6.2nm**	0.05	2.2	0.41	16.54	0.55	375

Supplementary Notes

Supplementary Note 1: Calculation of Hydrogen Generation Apparent Quantum Yield

The measured rates were used to calculate the apparent quantum yield (QY), defined as:

$$QY = \frac{n_e}{n_p} * 100\% = \frac{2n_{H_2}}{n_p} * 100\% \qquad (1)$$

Where n_e is the number of electrons participating in reduction reaction, n_p is the number of photons absorbed, and n_{H_2} is the number of hydrogen molecules formed and measured by the GC analysis. n_p was extracted from the measured illumination intensity and irradiation time, taking into account the solution optical density at the irradiation wavelength, which was kept the same for all samples.

Supplementary Note 2: X-ray Photoelectron Spectroscopy (XPS) Analysis

XPS measurements show the characteristic binding energies (BE) of each element according to its compound and oxidation state. HNPs with different Au metal domain sizes were measured for Au 4*f* binding energy. Supplementary Figure 4a shows comparison of the detailed Au $4f_{7/2}$ and $4f_{5/2}$ spectra for CdS-Au HNPs with different Au metal domain sizes. In all samples the BE spectra shows a dominant doublet

consistent with the zero-valent Au ($84.0eV$) which designates the metallic nature of these domains, alongside with an oxidized Au^{+} doublet ($85.0eV$) contribution which is more prominent at the smaller Au domain HNPs. Supplementary Figure 4b present the normalized Au peak intensities which shows a pronounced increase in the Au peak intensity for increased metal domain size.

For each Au size domain sample, atomic concentration percent were obtained and Cd:Au ratio was calculated with respect to the ionization cross section. The results are summarized on Supplementary Table 2. This nearly 10 fold increased relative Au atomic concentration indicates the increase of the Au metal growth of the single tip on the CdS nanorod apex, while the relative amount of Cd atoms is showing minor changes. In consistence with the Au $4f$ BE intensities measured for the different Au sizes, the Cd:Au ratio is decreasing from 16.66 to 1.54 for 1.6nm to 6.2nm in diameter, respectively.

Similar to the described CdS absorption normalization presented in the manuscript, the hydrogen generation rate can be normalized to the Cd:Au ratio of each HNPs sample as presented in Supplementary Figure 5. The observed trend of non-monotonic behavior of hydrogen production rate and efficiency (black curve) is also revealed by this form of normalization (red curve). In this manner, the rate is normalized to Au the co-catalysts domain loading and therefore reveals complementary aspect of the metal co-catalysts size effect on the efficiency of photocatalytic hydrogen production.

Supplementary Note 3: Charge Carriers Dynamics and Fitting

TA decay curves monitoring bleach recovery of the HNPs with different metal domain sizes were fitted to a tri-exponential function. The TA decay curves of the NRs sample was fitted to a bi-exponential decay in Supplementary Table 3.

In the tri-exponential decay model fitting for HNPs, the third decay component is fairly long (above 370psec for all surface coatings). We assume that this component, which is also least accurately determined in our fits due to the limited sampled temporal window, is most likely not related to the electron transfer from the semiconductor component to the metal domain, but rather to electron-hole recombination. This is confirmed by the dynamics of the bare NRs domain, for which the long decay recovery is within the same range of the HNPs longer decays components. This elongation of the time constant is a result of the absence of a metal that serves as an electron sink, and therefore only semiconductor related decay routes

are possible here, such as radiative electron–hole recombination or non-radiative decay kinetics. We attribute the second decay components in general to the transfer of the excited electron to the metal domain, indicating increased effective electron transfer rates for increased Au domain sizes. This behavior is expressed both by the shortening of the excited electron lifetimes and the increased amplitude of the decay curves.

Power dependent TA measurements at 450nm excitation wavelength were done on all different Au tipped HNPs sizes. Linear regime is kept up to 50nJ in all of the samples, as can be seen in Supplementary Figure 6. A comparison between the plasmon (probed at 540nm) and exciton (probed at 450nm) response, shows quite substantial influence of the plasmon on the exciton signal due to the high absorption signal of the plasmon overlapping with the exciton bleach signal. Thus, the exciton intensity is a bit decreased by this overlap with plasmon absorption signal.

Supplementary Figure 7 present power dependent measurements at 590nm excitation wavelength, in the spectral region of the Au absorption solely, for different Au tipped HNPs sizes, showing increased relaxation times, due to electron-phonon interaction, of the hot SPR with increasing the power of the pump. This behavior, which is well known for metal nanoparticles, is much more obvious in the larger Au domains. The rise time in all measurements was found to be the same. Comparison of plasmon dynamics of the three different Au domain sizes reveals no size dependence on the plasmon bleach relaxation with measured decay times of 1.5-1.7ps, as can be seen in Supplementary Figure 8. These observations are consistent with previous reports.[1]

Supplementary Note 4: Kinetic Model

We propose a minimalistic model consisting of several kinetic steps as sketched in Supplementary Figure 9. The photo-excited electron in the semiconductor can relax to the co-catalyst metal domain (with a rate given by k_{ET}) or by electron-hole recombination (k_{e-h}). The latter is rather slow[2] compared to the other processes and is assumed to be constant independent of the Au tip size ($k_{e-h} = \frac{1}{5}\,\mathrm{nsec}^{-1}$). The electron on the metal tip can either promote water reduction reaction (k_{WR}) or undergo back recombination with the hole that is left behind on the semiconductor domain (k_{rec}), which is modeled by the hole transfer from the semiconductor to the metal tip. An additional important process is the trapping of the hole (k_{ST}) with a rate that is comparable to the electron transfer into the metal tip. Once the hole is trapped it

blocks the electron transfer channel by the electron-hole Coulomb interaction, which also leads to a localization of the electron.

The kinetic master equation for the population of the excited state on the CdS nanorods (P_{CdS^*}), ground state on the CdS nanorod (P_{CdS}), the electron on the Au tip (P_{Au}) and the water reduction (P_{H_2O/H_2}) are given by:

$$\frac{dP_{CdS^*}}{dt} = -(k_{ET} + k_{ST} + k_{e-h})P_{CdS^*} \quad (2)$$

$$\frac{dP_{CdS}}{dt} = -k_{eh}P_{CdS^*} + k_{rec}P_{Au} \quad (3)$$

$$\frac{dP_{Au}}{dt} = k_{ET}P_{CdS^*} - (k_{WR} + k_{rec})P_{Au} \quad (4)$$

$$\frac{dP_{H_2O/H_2}}{dt} = k_{WR}P_{Au} \quad (5)$$

The time evolution of these populations is given by:

$$P_{CdS^*}(t) = P_{CdS^*}(0)e^{-(k_{ET}+k_{ST}+k_{e-h})t} \quad (6)$$

$$P_{Au}(t) = \frac{k_{ET}}{k_{WR}+k_{rec}-(k_{ET}+k_{ST}+k_{e-h})}\left(e^{-(k_{ET}+k_{ST}+k_{e-h})t} - e^{-(k_{WR}+k_{rec})t}\right) \quad (7)$$

$$P_{H_2O/H_2}(t) = \frac{k_{ET}k_{WR}}{k_{WR}+k_{rec}-(k_{ET}+k_{ST}+k_{e-h})}\left(\frac{\left(1-e^{-(k_{ET}+k_{ST}+k_{e-h})t}\right)}{k_{ET}+k_{ST}+k_{e-h}} - \frac{\left(1-e^{-(k_{WR}+k_{rec})t}\right)}{k_{WR}+k_{rec}}\right) \quad (8)$$

The efficiency of this overall photocatalytic process is determent by the rate of the hydrogen generation P_{H_2O/H_2}, given by:

$$QY = P_{H_2O/H_2}(t \to \infty) = \frac{k_{ET}k_{WR}}{(k_{WR}+k_{rec})(k_{ET}+k_{ST}+k_{e-h})} \quad (9)$$

The semiconductor-metal charge transfer (k_{ET}) and the back recombination (k_{rec}) rates can be described by Auger processes, with rates determined by Fermi's golden rule:

$$k_{ET} = \frac{2\pi}{\hbar}\sum_f |t_e|^2 \delta(\varepsilon_c - \varepsilon_f) \quad (10)$$

where t_e is the electron tunneling matrix element (assumed to be independent of the Au tip radius, r), ε_c is the energy of the conduction band minimum and ε_f is the final of the electron on the metal tip. Assuming that t_e is independent of the final state f in the metal, we approximate the rate as (this simplification has already been employed previously for electron transfer in similar systems and showed good correlation to experimental results.[3,4]):

$$k_{ET} = \frac{2\pi}{\hbar}|t_e|^2 \sum_f \delta(\varepsilon_c - \varepsilon_f) = \frac{2\pi}{\hbar}|t_e|^2 \rho(\varepsilon_c) \quad (11)$$

Where the density of state $\rho(\varepsilon_c)$ in the metal tip is given by:

$$\rho(\varepsilon) = \frac{4\pi}{3} r^3 \frac{1}{2\pi^2}\left(\frac{2m_e^*}{\hbar^2}\right)^{\frac{3}{2}} \sqrt{\varepsilon} = \frac{2}{3\pi} r^3 \left(\frac{2m_e^*}{\hbar^2}\right)^{\frac{3}{2}} \sqrt{\varepsilon} \quad (12)$$

Substitution of 12 in 11 gives:

$$k_{ET} = \frac{4}{3\hbar} r^3 |t_e|^2 \left(\frac{2m_e^*}{\hbar^2}\right)^{\frac{3}{2}} \sqrt{\varepsilon_c + \phi(r) + \varepsilon_F} \quad (13)$$

where m_e^* is the effective mass of the electron in the metal tip, ε_F is the Fermi energy of the metal assumed independent of r, and $\phi(r)$ is the work function modeled by:[5,6]

$$\phi(r) = \phi_{bulk} - \frac{2\gamma v_M}{zFr} \quad (14)$$

where γ is the surface tension, v_M is the molar volume, z is number of transferred electrons, F is Faraday's constant, and as before r is the radius of the metal tip.

A similar approach for the back reaction, modeled as a hole transfer from the semiconductor to the metal tip yields:

$$k_{rec} = \frac{4}{3\hbar} r^3 |t_h|^2 \left(\frac{2m_h^*}{\hbar^2}\right)^{\frac{3}{2}} \sqrt{\varepsilon_v + \phi(r) + \varepsilon_F} \quad (15)$$

where m_h^* is the effective mass of the hole on the metal, ε_v is the energy of the top of the valance band, and t_h is the hole tunneling matrix element (assumed to be independent of r).

The water reduction reaction on the metal co-catalyst is described by the cathodic rate in the Butler-Volmer model for redox reactions.[7] The electron transfer process along is given by a Marcus-like expression:

$$k_{WR} = k_{WR}^0 \exp\left(-\frac{\alpha eF}{RT}\left(\varepsilon_W - \phi(r)\right)\right) \quad (16)$$

where k_{WR} is the electron reduction rate of an absorbed water molecule, k_{WR}^0 is the conditional rate constant, R is the gas constant, T is the temperature, α is the electron transfer coefficient which determines symmetry of the transition state (e.g., $\alpha = \frac{1}{2}$ corresponds to a transition state with equal contributions from the reactants and products), F is Faraday's constant, ε_W and $\phi(r)$ are the water reduction potential and the metal work-function, respectively. The anodic rate for the hydrogen oxidation (back reaction) can be neglected because the hydrogen concentration is small

compared to the proton concentration at the experimental pH level. In contrast to the charge and recombination rates, k_{WR} depends exponentially on size of the metal domain through the dependence of $\phi(r)$.

We now discuss the effect of adjusting the free parameters in the theory. The electron tunneling matrix element was determined by fitting the rate of the electron transfer to the metal component (k_{ET}) to the TA measurements bleach decay rate which proves to fit this rate term as presented in Figure 3C in the manuscript. t_e was found to be 5.6×10^{-5} eV which is in the range of previously reported values for overlap matrix parameter at similar nano-junction.[3] As mentioned above, the competitive routes, trapping or recombination were kept constant (without size dependency) and thier time constants $\tau_{ST} = k_{ST}^{-1}$ and $\tau_{e-h} = k_{e-h}^{-1}$ were taken to be 18psec and 5nsec, respectively. These values are also with in agreement with fast hole trapping and low efficient fluorescence known for CdS NRs. Investigation of the rest of the fitted parameters led to the next described values. The hole wave-function coupling, expressed as the hole tunneling matrix element, t_h, was found to be 1.1×10^{-6} eV. This value allows obtaining a back recombination time constant(τ_{rec}) in order of μsec for small Au tip as reported previously for similar systems[2] (half-life of 1.2 ± 0.6 μsec). Supplementary Figure 10 reveals the hole tunneling matrix element dependence of the efficiency model at given symmetry factor. Higher energies of the overlap parameter can predict lower efficiencies and non-monotonic behavior with optimum at smaller sizes of the metal co-catalyst than the measured efficiencies (black square), especially at higher electron transfer coefficient values.

Several reported values of surface tension for gold nanoparticles were tested to fit the suggested model.[5,8,9] Supplementary Figure 11 presents the efficiency dependence on the surface tension at given symmetry factor. Reasonable surface tension values (γ) are between 2.5-4 J m^{-1}. Higher surface tension values result in high work function values for the metal domain above the semiconductor conduction band minimum leading to vanishing electron injection rates. Furthermore, in larger tip sizes the efficiency is overestimated due to large over-potential relative to the standard water reduction potential at the experimental pH.

Another key parameter that was investigated for its influence on the model behavior is the standard rate constant, k_{WR}^{0}. Although this is usually an experimental factor depending strongly on the system features and conditions, values of this parameter

were tested between $1 \times 10^6 - 1 \times 10^8$ sec^{-1}. Higher or lower value would result in a very fast water reduction time (τ_{WR}) at sub-psec scale or very slow at the several second scale, respectively, both of which are no match for the predicted rates for such redox reaction. The conditional rate constant was found to be 1×10^8 sec^{-1}. Supplementary Figure 12 shows the efficiency dependence on the conditional rate constant at given symmetry factor. High values of this parameter lead to non-monotonic behavior with late optimum at larger size of the metal domain relatively to the experimental observations. In contrast, lower values by one order of magnitude do not reach the measured efficiencies.

In addition, from all different comparisons above, the value of the electron transfer coefficient, α, was set as 0.25, which is in the range of values reported in the literature for similar systems.[7] Higher values of the symmetry coefficient lead to a narrower non-monotonic behavior and much faster decay in the hydrogen production efficiency already at small radii of the co-catalyst relatively to the experimental efficiencies.

Supplementary Methods

Chemicals: Trioctylphosphine (TOP, Sigma Aldrich, 90%) was vacuum distilled before use and stored under inert atmosphere. All other chemicals were used as purchased: cadmium oxide (>99.99%), trioctylphosphine oxide (TOPO, 99%), 1-octadecene (ODE, technical grade, 90%), oleic acid (95%), Octadecylamine (ODA, ≥99%), Didodecyldimethyl ammonium bromide (DDAB, 98%) and Gold(III)chloride (99%), mercaptoundecanoic acid (MUA, 95%), mercaptohexanoic acid (MHA, 90%), mercaptopropionic acid (MPA, ≥99%), 2-mercaptoethanesulfonic acid (MSA, 98%), O-(2-Carboxyethyl)-O′-(2-mercaptoethyl)heptaethylene glycol (≥95%), L-glutathione reduced (GSH, ≥98.0%), sodium sulfide nonahydrate (98.0-100.5%), sodium sulfite (>98%), Polyethylenimine, (PEI, branched average Mw ~25,000), Poly(styrene-co-maleic anhydride), cumene terminated (PSMA) were purchased from Sigma Aldrich. Oleylamine (technical grade, 90%) was purchased from Across. Octadecylphosphonic acid (ODPA) and hexylphosphonic acid (HPA) were purchased from PCI Synthesis. Sulfur (>99.0%) was purchased from Merck.

Synthesis of CdS Seeds: CdS nanocrystal seeds were synthesized by a modification of a previously reported procedure.[10] cadmium oxide (CdO; 0.106g), oleic acid (OA; 2.26g) and 1-octadecene (ODE; 20g) were mixed in a 100ml three-neck flask. The mixture was heated to 100°C and placed under vacuum for 1 hour followed by purging

three times with argon. Under argon atmosphere, the solution was heated to 260°C to dissolve the CdO, forming a clear colorless solution. A precursor solution consisting of sulfur (0.013g) and ODE (7ml) was rapidly injected into the hot solution. The reaction time was typically 90sec for CdS seeds with diameter of ~3.1 nm. The reaction was quenched by removing the heating mantle and cooling with fan. The crude reaction mixture was precipitated with acetone followed by centrifugation. For further purification, the particles were dissolved in toluene and the precipitation procedure was repeated several times.

Synthesis of CdS Nanorods: CdS nanorods were synthesized by a modification of a previously reported procedure employing seeded growth.[11] CdO (0.12g), trioctylphosphine oxide (TOPO; 6.0g), octadecylphosphonic acid (ODPA; 0.68g) and hexylphosphonic acid (HPA; 0.04g) were mixed in a 100ml three-neck flask. The mixture was heated to 100°C and placed under vacuum for 1hour followed by purging three times with argon. Under argon atmosphere, the solution was heated to 350°C and at this temperature trioctylphosphine (TOP; 1.5ml) was injected into the hot solution. Next, the solution was heated further to 365°C, at which a precursor solution of CdS seeds (typically, $3x10^{-8}$ mol) and sulfur in TOP solution (1.6ml; 0.075g/ml) was rapidly injected into the hot solution. The temperature decreased and then recovered within 1-2min. The reaction time was 8min for 30x5 nm-sized CdS rods. The reaction was quenched by removing the heating mantle and cooling with fan. The crude reaction mixture was diluted with toluene. Methanol was added in order to precipitate the nanocrystals and remove excess surfactants. The NR samples had narrow size distribution according to their absorption spectra and TEM characterization measurements and statistics (Supplementary Figure 1).

Nanoparticle Characterization: TEM characterization was performed using a Tecnai T12 G^2 Spirit and Tecnai F20 G^2, respectively. All size statistics are done with "Scion image" program on 200 particles. Absorption was measured with a JASCO V-570 UV-vis-near IR spectrophotometer. Extinction coefficient values of the NRs were calculated using a previously reported method.[12]

Synthesis of CdS-Au Hybrid Nanorods: In order to obtain size control of the site-selective deposition of the Au domain on the CdS NRs, two different synthetic strategies were combined in a single procedure. In the first step, a precursor stock solution consisting of octadecylamine (ODA; 0.055g), di-dodecylammoniumbromide (DDAB; 0.021g) and $AuCl_3$ (0.015mg) in toluene (10ml) was sonicated for 15min to

dissolve the $AuCl_3$, and the solution changes color from dark brown to yellow. In order to achieve selective growth of 1.5-1.8 nm gold tips on one apex of the NRs, the molar ratio of used was 700-900 Au ions per NR depending on the specific properties of the rods. Diluted Au growth stock solution was added to CdS NRs (typically ~$7x10^{-9}$ mol) in toluene (20ml) in 50ml flask under flowing argon. The solutions are mixed and left for 1 hour in the dark at room temperature. The product hybrid nanoparticles (HNPs) are then washed and precipitated with acetone followed by separation via centrifugation.[13] Next, light-induced metal deposition step is conducted. A mixture of small tipped CdS-Au hybrid NRs (typically ~$8x10^{-10}$ mol) in 10ml toluene solution was placed under inert atmosphere and at low temperature environment, ~2-4°C. Different Au^{3+}/NR molar ratios, varying between 1000-15000 Au ions per NR, of the diluted Au growth stock solution were added to the mixture, followed by illumination with blue LED (100W) for typically 30 min. This step results in additional growth of the metal tip achieving CdS-Au HNPs with various metal sizes. The final product HNPs are then washed and precipitated with acetone followed by separation via centrifugation.

Phase transfer: For transfer of the HNPs to water, ligand exchange and polymer coating method were used. Polymer coating was done with Polyethylenimine (PEI) by mixing HNPs solution (1ml) with PEI (0.15g; MW 25,000) in chloroform (1ml) for 1 hour. Then the particles are precipitated and washed with cyclohexane (1:1 chloroform/cyclohexane), followed by centrifugation. TDW is added to the precipitate and residues of PEI are removed by centrifugation. In addition, polymer-coated particles are washed again through 100KDa cellulose membrane, to remove polymer residues in the water suspended hybrids.

Hydrogen evolution rate and efficiency measurements: In order to determine and measure the evolved hydrogen gas from the photocatalytic reaction using the HNP model systems, the following set-up is used. The photocatalysts were dispersed in TDW solution (2ml; optical density, OD ~1 at 405 nm). The photocatalyst solution was placed in a quartz cuvette and hole scavengers, Na_2S-$9H_2O$ and Na_2SO_3, (typically 0.05M and 0.07M respectively), were added to the water. The solution is purged with argon for 20min and stirred. The HNPs were then illuminated with 28mW 405 nm laser, producing 5.7×10^{16} photon sec^{-1}. Aliquots of the reaction vessel head space were taken using a gas tight syringe at different time intervals and

detected and quantified using Varian gas chromatograph (model 6820) equipped with a molecular sieve (5Å) packed column and a thermal conductivity detector. The resulting chromatograms and hydrogen concentration are obtained by the comparison to a calibration curve of known hydrogen amounts.

Inductively coupled plasma mass spectrometry measurements (ICP-MS): For ICP-MS, following hydrogen generation kinetic measurements a 100ml of HNPs solution was etched overnight in 1 mL of 69% nitric acid. Following sonication, 100 μL of the HNPs solution was mixed with 3.35 mL of TDW and analyzed by ICP-MS (cx7500, Agilent) for Cd. The quantity of Cd in each solution was calculated using external calibration with standard Cd solutions.

X-ray Photoelectron Spectroscopy Measurements (XPS): XPS measurements were performed with a Kratos AXIS Ultra X-ray photoelectron spectrometer (Kratos Analytical, Manchester, UK). Spectra were acquired using the Al-Kα monochromatic X-ray source ($1486.7eV$) with 0° take of angle (normal to analyzer). The vacuum pressure in the analyzing chamber was maintained at $\sim 2 \times 10^{-9}\, Torr$ during the acquisition process. The survey spectra were collected with pass energy$160eV$, and $0.5eV$ step size, dwell time $250ms$. High resolution XPS spectra were collected for Cd 3*d*, S 2*p*, C 1*s* and Au 4*f* peaks, with pass energy $20eV$ and $0.1eV$ step size, dwell time $1000ms$. Data analyses were done using Kratos Vision (Kratos Analytical Ltd.) processing software and Casa XPS (Casa Software Ltd.). The binding energies were calibrated using C 1s peak energy as $285.0eV$.[14]

Transient Absorption Measurements: The laser system employed for ultrafast transient absorption (TA) was based on a Ti-Sapphire chirped pulse amplified source, with maximum output energy of about 800 μJ, 1 kHz repetition rate, central wavelength of 800 nm and pulse duration of about 150 fs. Excitation pulses at 400 nm were obtained by doubling the fundamental frequency in a β-Barium borate (BBO) crystal while other pump photons at different wavelength were generated by non-collinear optical parametric amplification in BBO, with pulse duration around 100 fs. Pump pulses were focused to 175 μm diameter spot. Probing was achieved in the visible range by using white light generated in a thin sapphire plate, and in the UV-visible range by using a thin Calcium Fluoride plate. Chirp-free transient absorption spectra were collected by using a fast optical multichannel analyzer (OMA) with

dechirping algorithm. The measured quantity is the normalized transmission change, $\Delta T/T$.

Supplementary References

1. Link, S. & El-Sayed, M.A. Spectral properties and relaxation dynamics of surface plasmon electronic oscillations in gold and silver nanodots and nanorods. *J Phys Chem B* **103,** 8410-8426 (1999).
2. Wu, K.F., Zhu, H.M., Liu, Z., Rodriguez-Cordoba, W. & Lian, T.Q. Ultrafast Charge Separation and Long-Lived Charge Separated State in Photocatalytic CdS-Pt Nanorod Heterostructures. *J Am Chem Soc* **134,** 10337-10340 (2012).
3. Tvrdy, K., Frantsuzov, P.A. & Kamat, P.V. Photoinduced electron transfer from semiconductor quantum dots to metal oxide nanoparticles. *P Natl Acad Sci USA* **108,** 29-34 (2011).
4. Stockwell, D. *et al.* Comparison of Electron-Transfer Dynamics from Coumarin 343 to TiO2, SnO2, and ZnO Nanocrystalline Thin Films: Role of Interface-Bound Charge-Separated Pairs. *Journal of Physical Chemistry C* **114,** 6560-6566 (2010).
5. Plieth, W.J. Electrochemical Properties of Small Clusters of Metal Atoms and Their Role in Surface Enhanced Raman-Scattering. *J Phys Chem-Us* **86,** 3166-3170 (1982).
6. Redmond, P.L., Hallock, A.J. & Brus, L.E. Electrochemical Ostwald ripening of colloidal Ag particles on conductive substrates. *Nano Lett* **5,** 131-135 (2005).
7. Zhao, J., Holmes, M.A. & Osterloh, F.E. Quantum Confinement Controls Photocatalysis: A Free Energy Analysis for Photocatalytic Proton Reduction at CdSe Nanocrystals. *ACS Nano* **7,** 4316-4325 (2013).
8. Jiang, Q., Liang, L.H. & Zhao, D.S. Lattice contraction and surface stress of fcc nanocrystals. *J Phys Chem B* **105,** 6275-6277 (2001).
9. Nanda, K.K., Maisels, A. & Kruis, F.E. Surface tension and sintering of free gold nanoparticles. *J Phys Chem C* **112,** 13488-13491 (2008).
10. Yu, W.W. & Peng, X.G. Formation of high-quality CdS and other II-VI semiconductor nanocrystals in noncoordinating solvents: Tunable reactivity of monomers. *Angewandte Chemie-International Edition* **41,** 2368-2371 (2002).

11. Carbone, L. *et al.* Synthesis and micrometer-scale assembly of colloidal CdSe/CdS nanorods prepared by a seeded growth approach. *Nano Lett* **7,** 2942-2950 (2007).
12. Shaviv, E., Salant, A. & Banin, U. Size Dependence of Molar Absorption Coefficients of CdSe Semiconductor Quantum Rods. *Chemphyschem* **10,** 1028-1031 (2009).
13. Menagen, G. *et al.* Selective Gold Growth on CdSe Seeded CdS Nanorods. *Chemistry of Materials* **20,** 6900-6902 (2008).
14. Moulder, J.F. & Chastain, J., *Handbook of X-ray Photoelectron Spectroscopy: A Reference Book of Standard Spectra for Identification and Interpretation of XPS Data*. (Physical Electronics, 1995).
